# Small cell lung carcinoma cell line screen of etoposide/carboplatin plus a third agent

**DOI:** 10.1002/cam4.1131

**Published:** 2017-08-01

**Authors:** Beverly A. Teicher, Thomas Silvers, Michael Selby, Rene Delosh, Julie Laudeman, Chad Ogle, Russell Reinhart, Ralph Parchment, Julia Krushkal, Dmitriy Sonkin, Larry Rubinstein, Joel Morris, David Evans

**Affiliations:** ^1^ Developmental Therapeutics Program Division of Cancer Treatment and Diagnosis National Cancer Institute Bethesda Maryland 20892; ^2^ Molecular Pharmacology Group Leidos Biomedical Research, Inc. Frederick National Laboratory for Cancer Research Frederick Maryland 21702; ^3^ Biometric Research Program Division of Cancer Treatment and Diagnosis Bethesda Maryland 20892

**Keywords:** SCLC, SCLC cell‐based screen, SCLC combination screen, Small cell lung cancer

## Abstract

The SCLC combination screen examined a 9‐point concentration response of 180 third agents, alone and in combination with etoposide/carboplatin. The predominant effect of adding a third agent to etoposide/carboplatin was additivity. Less than additive effects occurred frequently in SCLC lines sensitive to etoposide/carboplatin. In SCLC lines with little or no response to etoposide/carboplatin, greater than additive SCLC killing occurred over the entire spectrum of SCLC lines but never occurred in all SCLC lines. Exposing SCLC lines to tubulin‐targeted agents (paclitaxel or vinorelbine) simultaneously with etoposide/carboplatin resulted primarily in less than additive cell killing. As single agents, nuclear kinase inhibitors including Aurora kinase inhibitors, Kinesin Spindle Protein/EG5 inhibitors, and Polo‐like kinase‐1 inhibitors were potent cytotoxic agents in SCLC lines; however, simultaneous exposure of the SCLC lines to these agents along with etoposide/carboplatin, generally, resulted in less than additive cell killing. Several classes of agents enhanced the cytotoxicity of etoposide/carboplatin toward the SCLC lines. Exposure of the SCLC lines to the MDM2 inhibitor JNJ‐27291199 produced enhanced killing in 80% of the SCLC lines. Chk‐1 inhibitors such as rabusertib increased the cytotoxicity of etoposide/carboplatin to the SCLC lines in an additive to greater than additive manner. The combination of GSK‐3*β* inhibitor LY‐2090314 with etoposide/carboplatin increased killing in approximately 40% of the SCLC lines. Exposure to the BET bromodomain inhibitor MK‐8628 increased the SCLC cell killing by etoposide/carboplatin in 20–25% of the SCLC lines. Only 10–15% of the SCLC lines had an increased response to etoposide/carboplatin when simultaneously exposed to the PARP inhibitor talazoparib.

## Introduction

The standard of care for limited‐stage and extensive‐stage small cell lung carcinoma (SCLC) has not changed in more than 30 years. In the US, the standard of care for newly diagnosed SCLC is etoposide and a platinum complex (cisplatin or carboplatin) administered as systemic chemotherapy 1. The scheduling of etoposide and platinum as well as the doses and dose intensities of each drug have been varied without improvement in survival 2, 3. Many drugs and investigational agents have been added to etoposide and platinum without clinical success 4, 5. Initially, 60–80% of SCLC patients responded to etoposide and a platinum complex; however, recurrent SCLC is highly therapeutically resistant. SCLC was named in the Recalcitrant Cancer act by the US Congress because the survival rates are dismal and little research effort has been directed toward improving the treatment outcome for SCLC patients 6.

Recurrent SCLC has proven to be resistant to many therapeutics, therefore, one strategy may be improving the therapeutic efficacy of front‐line therapy. This study reports findings from a combination screen of 63 human SCLC lines exposed to etoposide/carboplatin plus a third agent to identify compounds which may be added to the current standard of care to therapeutic advantage.

## Materials and Methods

### Cell lines

The 63 small cell lung carcinoma (SCLC) cell lines used in the study were purchased from American Type Culture Collection (ATCC, Manassas, VA) or were obtained from the NCI repository. In addition, NCI‐H28 mesothelioma, NCI‐H2066 mixed SCLC/NSCLC NCI‐H1650 NSCLC, and A549 NSCLC purchased from ATCC were included as comparators to the SCLC lines. Cells were maintained in a 5% CO_2_ ‐humidified incubator at 37°C. The morphology for each cell line along with the patient prior treatment and response to treatment, where known have been described previously 6. The SCLC lines were authenticated using the Applied Biosystems Identifiler kit for short tandem repeat analysis (15 loci). The lines were thawed from the banked stock and samples were taken for Identifiler analysis within passages 2–5. New cells from the same frozen stock were thawed after a maximum of 20 passages, which did not exceed five continual months in culture. The human A549 NSCLC line was run on each plate as a screen control.

### Compounds

The SCLC lines were screened with a selection of 180 compounds from the FDA‐approved anticancer drugs (available from NCI at: http://dtp.nci.nih.gov/branches/dscb/oncology_drugset_explanation.html) and a library of >500 investigational oncology small molecules, alone and in combination with etoposide (0.3 *μ*mol/L) and carboplatin (3.7 *μ*mol/L).

### Screen

Twelve lines (11 SCLC and A549 human NSCLC cell line control) were screened per run. Each of the 12 lines was grown and harvested using standard tissue culture procedures. On day 1, the cells were collected and suspended at the desired density in 300 ml of media and plated (Tecan Freedom Evo). The cell inoculum was added to 384‐well plates (15 test plates, 1 control plate) (CulturPlates, PE, Waltham, MA). After cell inoculation, the plates were moved to a humidified 37°C incubator with 5% CO_2_. Compounds were added 24 h after initial cell plating. Each compound was tested at nine concentrations alone or with etoposide (0.3 *μ*mol/L)/carboplatin (3.7 *μ*mol/L) with a final DMSO concentration of 0.25%. After compound addition, the plates were returned to the humidified 37°C incubator for 96 h incubation. The positive controls were: topotecan (10 *μ*mol/L); doxorubicin (10 *μ*mol/L); tamoxifen (200 *μ*mol/L); and DMSO (0.25%). The incubation was terminated by adding ATP Lite (Perkin Elmer Inc., Waltham, MA) to each well, then reading the luminescence. All assays were performed in triplicate and wells around the outside edge of the plate were avoided. Results for the examples presented were confirmed.

### Data analysis

#### Analysis of drug response

To monitor the statistical validity of the data from the screen, the Z′ factor for each plate in the assay was calculated 7. A Z′ value of >0.5 is representative of a high‐quality assay. Concentration response data were fit with a 4‐parameter curve fit and IC_50_s determined as the median of three replicates.

#### Gene expression analysis

mRNA measurements using Affymetrix GeneChip^®^ Human Exon 1.0 ST Arrays (NCBI GEO accession number GSE73160), and experimental and computational procedures for mRNA data collection, processing, quality, and data normalization were reported previously 6. mRNA data were normalized using Robust Multi‐Array Average (RMA) and summarized at exon probeset and whole transcript level using AROMA. Normalized mRNA data were adjusted for batch effects using the ComBat function of the sva package 8, with adjustment confirmed by hierarchical sample clustering using the hclust function of R v. 3.3.0. Both whole transcript and exon data were analyzed for Pearson's correlation with drug response. Significance was evaluated using the Benjamini–Hochberg correction for false discovery rate (FDR) using *P*‐values from correlation tests of 18,690 transcripts or 284,258 exon probesets and 180 agents or agent combinations with etoposide/carboplatin 9. All *P*‐values were adjusted according to the FDR procedure.

Gene set expression analysis to identify proteins associated with response (log IC_50_) to talazoparib with etoposide/carboplatin in SCLC lines expressing SLFN11 (log_2_ SLFN11 transcript >6.4) was performed using BRB‐ArrayTools v.4.5.1 Gene Set Expression Comparison kit. The highest ranking Gene Ontology gene sets (*P* < 0.005) were significant by permutation tests using LS and Kolmogorov–Smirnov (KS) summary statistics, and Efron‐Tibshirani GSA maxmean test 10, 11, 12, 13.

#### Evaluation of response for an agent plus etoposide/carboplatin

Cellular response to combinations was based on Bliss independence 14. Cell growth was normalized as a fraction of control growth. The growth (*G*) of SCLC cells exposed to an agent in combination with etoposide/carboplatin was approximated using a linear model:

$$\text{log}G_{\mathrm{ti}}=\text{log}G_{\mathrm{si}}+\text{log}G_{\mathrm{ec}}+I_{\mathrm{ti}}$$

where *G*_ti_ is the proportion of cell growth with a combination at concentration i of the single agent, *G*_si_ is the proportion of cell growth with a single agent at that concentration, *G*_ec_ is the proportion of cell growth with etoposide/carboplatin, and *I*_*ti*_ is the interaction between the agent at concentration i and etoposide/carboplatin. The interaction effect was therefore estimated using a geometric sum across all nine concentrations as:

$$I_{t}=\frac{1}{9}\sum_{i=1}^{9}\left(\text{log}G_{\mathrm{ti}}\text{‐}\text{log}G_{\mathrm{si}}\text{‐}\text{log}G_{\mathrm{ec}}\right)$$

The median value of *I*_t_ was inferred for each SCLC line‐drug pair from multiple replicates. *I*_t_ was negative for synergistic interactions and positive for antagonistic interactions. Combinations among the highest 10% (>0.326) of *I*_t_ values were less than additive (antagonistic). Combinations among the lowest 10% (<−0.163) of *I*_t_ values were greater than additive (synergistic). Combinations among the 10–90% of the *I*_t_ values were additive.

## Results

**Figure 1:**
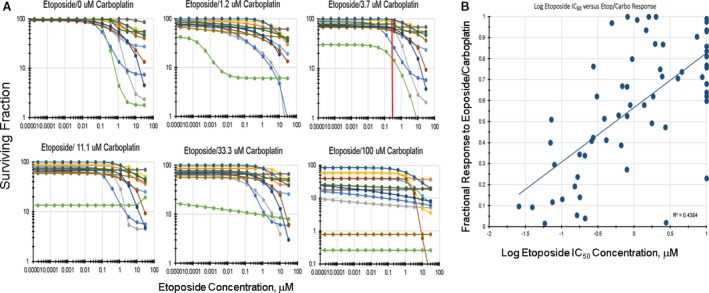
(A) Concentration response curves for 12 SCLC lines selected to have a range of response to etoposide. The graphs show 14‐point concentration response curves for: etoposide alone; etoposide plus 1.2 *μ*mol/L carboplatin; etoposide plus 3.7 *μ*mol/L carboplatin; etoposide plus 11.1 *μ*mol/L carboplatin; etoposide plus 33.3 *μ*mol/L carboplatin, and etoposide plus 100 *μ*mol/L carboplatin. The red line drawn at 0.3 *μ*mol/L etoposide plus 3.7 *μ*mol/L carboplatin indicates the standard of care concentrations selected for the combination screen. (B) Relationship between the IC_50_ concentrations for the 63 SCLC lines and the response of each SCLC line to etoposide (0.3 *μ*mol/L)/carboplatin (3.7 *μ*mol/L) (*R* ^2^ = 0.4384).

The screen examined a 9‐point concentration response of each of 180 third agents representing varied mechanisms of action, alone and in combination with etoposide/carboplatin and examined the data for additivity. The method for selection of the single concentrations for etoposide and carboplatin involved 12 SCLC lines with a range of response to etoposide alone. First, the SCLC lines were tested over a 7‐log range of concentrations to etoposide and then carboplatin was added in concentrations from 0 to 100 *μ*mol/L (Fig. 1A). The goal was to identify single concentrations of etoposide and carboplatin that in combination did not produce SCLC kill which would prohibit observation of additive or greater than additive SCLC cytotoxicity when a third agent was added. The concentrations which fulfilled the requirement were 0.3 *μ*mol/L etoposide plus 3.7 *μ*mol/L carboplatin (Fig. 1A).

The IC_50_ response of the 63 SCLC lines to etoposide spans 2.5 logs 6. The response of the SCLC lines to 0.3 *μ*mol/L etoposide plus 3.7 *μ*mol/L carboplatin spans 1 log (Fig. 1B). The log IC_50_ of etoposide plotted versus the response to 0.3 *μ*mol/L etoposide plus 3.7 *μ*mol/L carboplatin resulted in a line with *R* ^2^ = 0.4384 suggesting that both etoposide and carboplatin are active in the combination.

The predominant effect of adding a third agent to etoposide/carboplatin was additivity (Fig. S1). Interestingly, less than additive effects occurred more frequently in SCLC lines sensitive to etoposide/carboplatin than in SCLC lines with little or no response to etoposide/carboplatin. Greater than additive SCLC killing occurred over the entire spectrum of SCLC lines but never occurred in all the SCLC lines (Fig. S1). The greater than additive combination effects were more scattered. In no case did a third agent produce greater than additive effects in combination with etoposide/carboplatin with every SCLC line.

**Figure 2:**
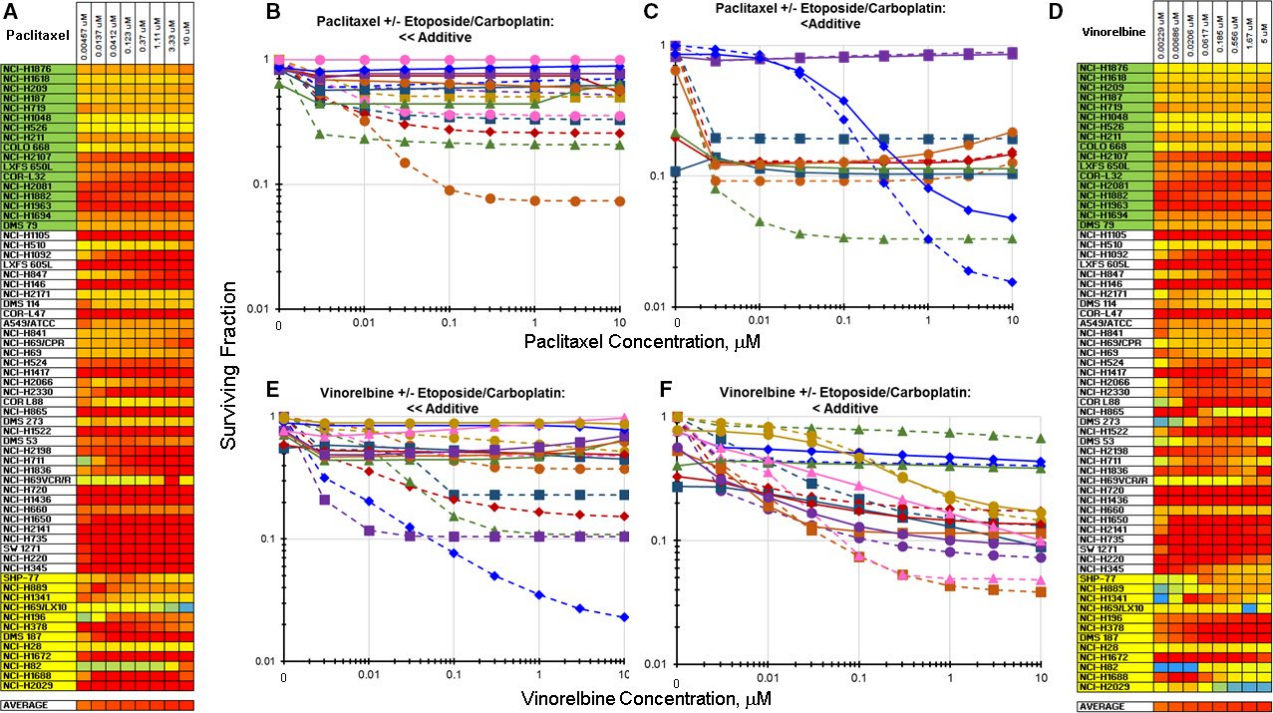
Heatmap and concentration response curves for the addition of paclitaxel or vinorelbine to etoposide/carboplatin in the SCLC lines. (A) Heatmap with the 63 SCLC lines listed from least responsive to most responsive to etoposide. Yellow indicates additivity of paclitaxel with etoposide/carboplatin. *I*_t_ estimated interaction of paclitaxel with etoposide/carboplatin 0.323 ± 0.256. Red indicates less than additive response to the combination of paclitaxel with etoposide/carboplatin. (B) Concentration response curves for representative SCLC lines exposed to paclitaxel alone (dotted lines), or to the combination of paclitaxel with etoposide (0.3 *μ*mol/L) and carboplatin (3.7 *μ*mol/L) (solid lines) where the combination was ≪additive. (C) Concentration response curves for representative SCLC lines exposed to paclitaxel alone (dotted lines), or to the combination of paclitaxel with etoposide (0.3 *μ*mol/L) and carboplatin (3.7 *μ*mol/L) (solid lines) where the combination was <additive. The color of the lines (dotted and solid) indicate the same SCLC line. (D) Heatmap with the 60 SCLC lines listed from least responsive to most responsive to etoposide. Yellow indicates additivity of vinorelbine with etoposide/carboplatin (*I*_t_ = 0.409 ± 0.322). Red indicates less than additive response to the combination of vinorelbine with etoposide/carboplatin. (E) Concentration response curves for representative SCLC lines exposed to vinorelbine alone (dotted lines), or to the combination of vinorelbine with etoposide (0.3 *μ*mol/L) and carboplatin (3.7 *μ*mol/L) (solid lines) where the combination was ≪additive. (F) Concentration response curves for representative SCLC lines exposed to vinorelbine alone (dotted lines), or to the combination of vinorelbine with etoposide (0.3 *μ*mol/L) and carboplatin (3.7 *μ*mol/L) (solid lines) where the combination was <additive. The color of the lines (dotted and solid) indicate the same SCLC line.

As a single agent, the tubulin stabilizer, paclitaxel, is a potent cytotoxic agent in many SCLC lines, although there were a few SCLC lines that were resistant to paclitaxel (Fig. S2). Exposing SCLC lines to paclitaxel simultaneously with etoposide/carboplatin resulted primarily in less than additive cell killing (Fig. 2A). While the SCLC lines were responsive to paclitaxel over the concentration range tested, when combined with exposure to etoposide/carboplatin, the killing of the SCLC lines decreased. For example, the IC_50_ for the SW1271 SCLC line exposed to paclitaxel was 0.0026 *μ*mol/L; however, the combination of paclitaxel with etoposide/carboplatin did not reach an IC_50_ at 10 *μ*mol/L paclitaxel, the highest concentration tested (Fig. 2B). In other SCLC lines, such as SHP77, the IC_50_ for paclitaxel alone (0.073 *μ*mol/L) and for the combination of paclitaxel with etoposide/carboplatin (0.057 *μ*mol/L) were quite similar (Fig. 2C).

As a single agent, the tubulin fragmenter, vinorelbine, was also a potent cytotoxic agent in the SCLC lines but produced less than additive cell killing when combined with etoposide/carboplatin (Fig. 2D). With many SCLC lines exposure to vinorelbine simultaneously with etoposide/carboplatin decreased cell killing compared with vinorelbine alone. The IC_50_ for vinorelbine as a single agent was 0.0023 *μ*mol/L for the SCLC line NCI‐H1436, while the IC_50_ for the combination of vinorelbine with etoposide/carboplatin in the NCI‐H1436 line was >10 *μ*mol/L (Fig. 2E). In other SCLC lines, such as NCI‐H660, the IC_50_s for single‐agent vinorelbine exposure (0.062 *μ*mol/L) and exposure to the combination of vinorelbine and etoposide/carboplatin (0.05 *μ*mol/L) were very similar (Fig. 2F).

**Figure 3:**
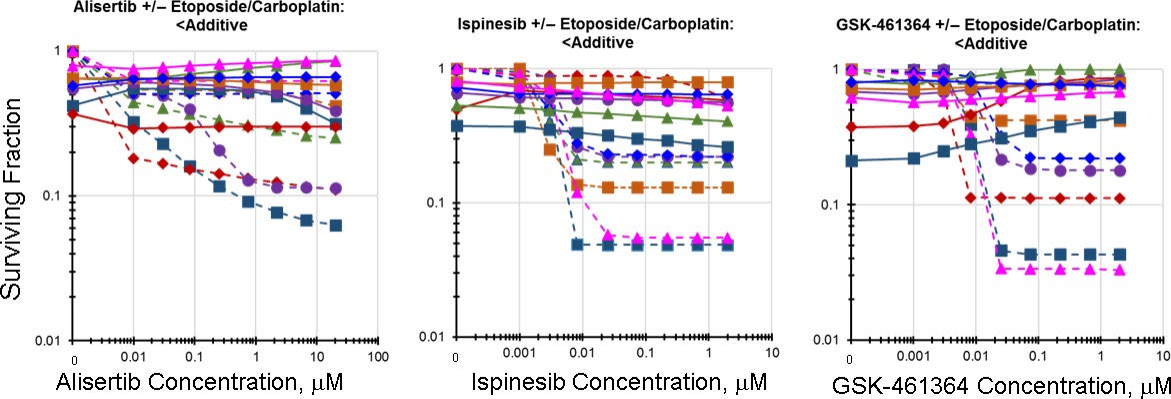
Concentration response curves for the addition of a nuclear kinase inhibitor to etoposide/carboplatin in the SCLC lines. Left: Concentration response curves for representative SCLC lines exposed to aurora kinase inhibitor, alisertib, alone (dotted lines), or to the combination of alisertib with etoposide (0.3 *μ*mol/L) and carboplatin (3.7 *μ*mol/L) (solid lines) (*I*_t_ = 0.298 ± 0.330). The color of the lines (dotted and solid) indicate the same SCLC line. Center: Concentration response curves for representative SCLC lines exposed to KSP/EG5 inhibitor, ispinesib, alone (dotted lines), or to the combination of ispinesib with etoposide (0.3 *μ*mol/L) and carboplatin (3.7 *μ*mol/L) (solid lines) (*I*_t_ = 0.328 ± 0.259). The color of the lines (dotted and solid) indicate the same SCLC line. Right: Concentration response curves for representative SCLC lines exposed to PLK1 inhibitor, GSK‐461364, alone (dotted lines), or to the combination of GSK‐461364 with etoposide (0.3 *μ*mol/L) and carboplatin (3.7 *μ*mol/L) (solid lines) (*I*_t_ = 0.424 ± 0.350). The color of the lines (dotted and solid) indicate the same SCLC line.

As single agents, nuclear kinase inhibitors including Aurora kinase inhibitors, Kinesin Spindle Protein/EG5 inhibitors, and Polo‐like kinase‐1 inhibitors were potent cytotoxic agents in SCLC lines that did not respond to etoposide (Fig. S2) 6. However, exposure of the SCLC lines to these agents, along with etoposide/carboplatin, generally, resulted in less than additive cell killing (Fig. S3). Exposure of the SCLC line NCI‐H1105 to single agent‐alisertib resulted in an IC_50_ of <0.01 *μ*mol/L, while the simultaneous exposure of NCI‐H1105 cells to alisertib along with etoposide/carboplatin resulted in an IC_50_ of 1.7 *μ*mol/L (Fig. 3). Ispinesib is a potent KSP/EG5 inhibitor. Exposure to single‐agent ispinesib killed the SCLC line NCI‐H1436 with an IC_50_ of 0.02 *μ*mol/L and simultaneous exposure to ispinesib along with etoposide/carboplatin resulted in an IC_50_ of >2 *μ*mol/L (Fig. 3). The PLK‐1 inhibitor GSK‐461364, as a single agent, potently killed the SCLC line NCI‐H1836 producing an IC_50_ of 0.006 *μ*mol/L. The simultaneous exposure to GSK‐461364 in combination with etoposide/carboplatin resulted in an IC_50_ of >2 *μ*mol/L (Fig. 3).

**Figure 4:**
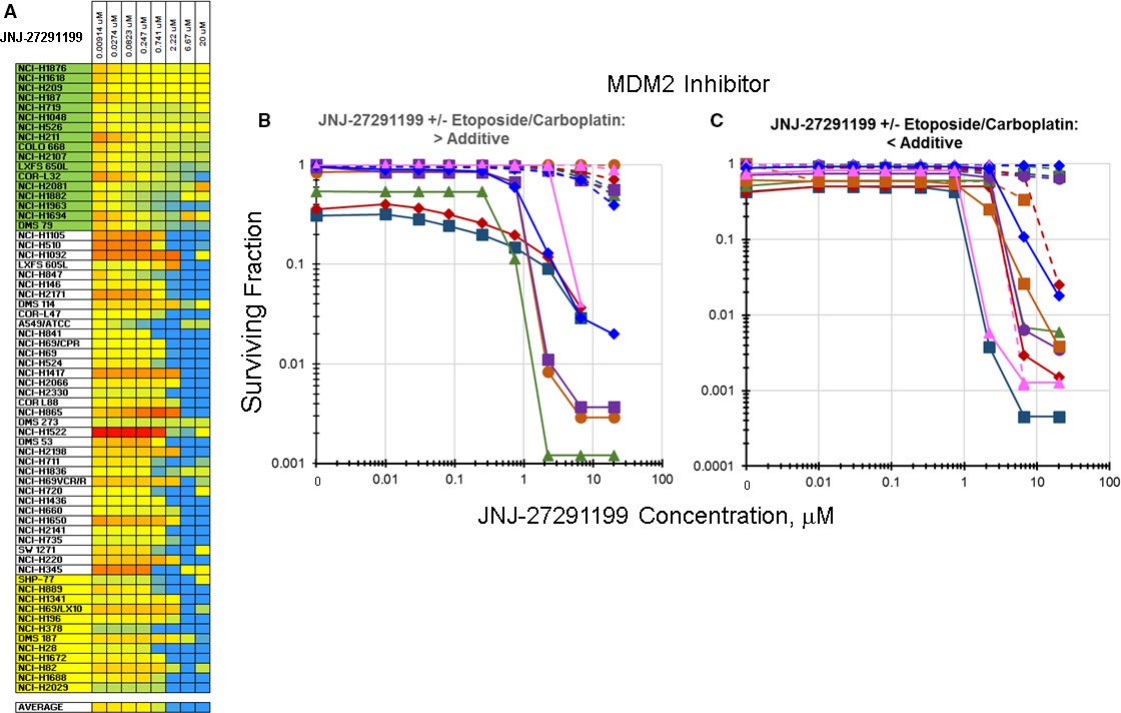
Heatmap and concentration response curves for the addition of the MDM2 inhibitor, JNJ‐27291199, to etoposide/carboplatin in the SCLC lines (*I*_t_ = −0.423 ± 0.273). (A) Heatmap with the 63 SCLC lines listed from least responsive to most responsive to etoposide. Blue indicates greater than additive response to the combination of JNJ‐27291199 with etoposide/carboplatin. Yellow indicates additivity of JNJ‐27291199 with etoposide/carboplatin. Red indicates less than additive response to the combination of JNJ‐27291199 with etoposide/carboplatin. (B) Concentration response curves for representative SCLC lines exposed to JNJ‐27291199 alone (dotted lines), or to the combination of JNJ‐27291199 with etoposide (0.3 *μ*mol/L) and carboplatin (3.7 *μ*mol/L) (solid lines) that produced greater than additive cell killing. C. Concentration response curves for representative SCLC lines exposed to JNJ‐27291199 alone (dotted lines), or to the combination of JNJ‐27291199 with etoposide (0.3 *μ*mol/L) and carboplatin (3.7 *μ*mol/L) (solid lines) that produced primarily additive cell killing. The color of the lines (dotted and solid) indicate the same SCLC line.

Several classes of agents enhanced the cytotoxicity of etoposide/carboplatin toward the SCLC lines (Fig. S4). Exposure to the MDM2 inhibitors as single agents produced little or no alteration in the proliferation of the SCLC lines (Fig. 4A). The MDM2 inhibitor JNJ‐27291199, although less potent than more recent MDM2 inhibitors, produced the same pattern of response in the NCI60 panel as other MDM2 inhibitors (Fig. S5). Exposure of the SCLC line NCI‐H840 to the MDM2 inhibitor JNJ‐27291199 produced an IC_50_ of >20 *μ*mol/L. Exposure of the NCI‐H840 SCLC line to JNJ‐27291199 along with etoposide/carboplatin resulted in an IC_50_ of 0.27 *μ*mol/L (Fig. 4B). Similarly, exposure of the SCLC line NCI‐H1105 to JNJ‐27291199 as a single agent resulted in an IC_50_ of >20 *μ*mol/L, while simultaneous exposure to JNJ‐27291199 and etoposide/carboplatin produced an IC_50_ of 0.01 *μ*mol/L (Fig. 4C).

**Figure 5:**
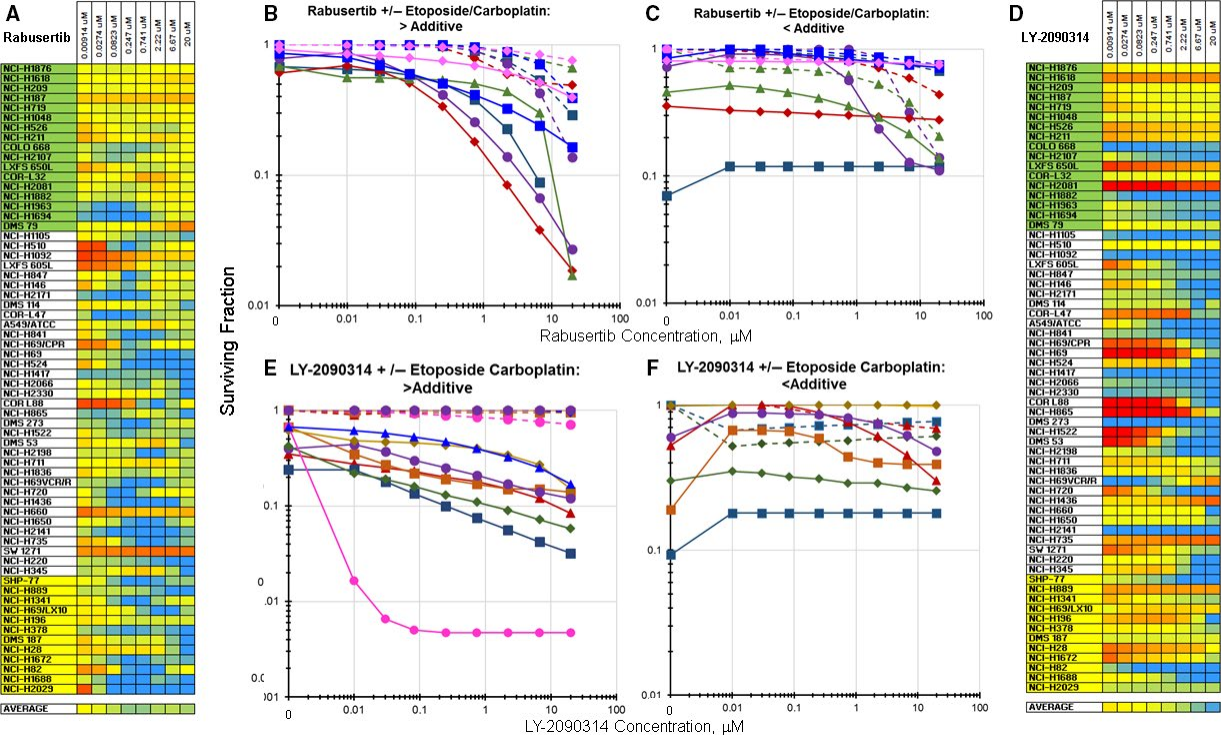
Heatmap and concentration response curves for the addition of the Chk1 inhibitor, rabusertib, or the GSK3*β* inhibitor, LY‐2090314, to etoposide/carboplatin in the SCLC lines. A. Heatmap with the 63 SCLC lines listed from least responsive to most responsive to etoposide. Blue indicates greater than additive response to the combination of rabusertib with etoposide/carboplatin (*I*_t_ = −0.181 ± 0.175). Yellow indicates additivity of rabusertib with etoposide/carboplatin. Red indicates less than additive response to the combination of rabusertib with etoposide/carboplatin. (B) Concentration response curves for representative SCLC lines exposed to rabusertib alone (dotted lines), or to the combination of rabusertib with etoposide (0.3 *μ*mol/L) and carboplatin (3.7 *μ*mol/L) (solid lines) that produced greater than additive cell killing. (C) Concentration response curves for representative SCLC lines exposed to rabusertib alone (dotted lines), or to the combination of rabusertib with etoposide (0.3 *μ*mol/L) and carboplatin (3.7 *μ*mol/L) (solid lines) that produced primarily additive cell killing. The color of the lines (dotted and solid) indicate the same SCLC line. (D) Heatmap with the 63 SCLC lines listed from least responsive to most responsive to etoposide. Blue indicates greater than additive response to the combination of LY‐2090314 with etoposide/carboplatin (*I*_t_ = −0.105 ± 0.273). Yellow indicates additivity of LY‐2090314 with etoposide/carboplatin. Red indicates less than additive response to the combination of LY‐2090314 with etoposide/carboplatin. (E) Concentration response curves for representative SCLC lines exposed to LY‐2090314 alone (dotted lines), or to the combination of LY‐2090314 with etoposide (0.3 *μ*mol/L) and carboplatin (3.7 *μ*mol/L) (solid lines) that produced greater than additive cell killing. (F) Concentration response curves for representative SCLC lines exposed to LY‐2090314 alone (dotted lines), or to the combination of LY‐2090314 with etoposide (0.3 *μ*mol/L) and carboplatin (3.7 *μ*mol/L) (solid lines) that produced primarily additive cell killing. The color of the lines (dotted and solid) indicate the same SCLC line.

Chk‐1 inhibitors such as rabusertib increased the cytotoxicity of etoposide/carboplatin when applied in simultaneous combination to the SCLC lines in an additive to greater than additive manner (Fig. 5A). The combination of rabusertib with etoposide/carboplatin increased the killing of the SCLC line NCI‐H524. Exposure of the NCI‐H524 SCLC line to rabusertib as a single agent resulted in an IC_50_ of 11.4 *μ*mol/L, while exposure to rabusertib simultaneously with etoposide/carboplatin resulted in an IC_50_ of 0.82 *μ*mol/L (Fig. 5B). As another example, exposure of the SCLC line DMS 79 to rabusertib as a single agent produced an IC_50_ of 13 *μ*mol/L. Adding rabusertib simultaneously to exposure to etoposide/carboplatin resulted in an IC_50_ of <0.01 *μ*mol/L (Fig. 5C).

The GSK‐3*β* inhibitor LY‐2090314 was tested in the screen. SCLC killing was increased in approximately 40% of the SCLC lines with the addition of LY‐2090314 to the combination of etoposide/carboplatin (Fig. 5D). Exposure of the SCLC line COLO668 to LY‐2090314 as a single agent produced an IC_50_ of >20 *μ*mol/L, while exposure of the COLO668 SCLC line simultaneously to LY‐2090314 along with etoposide/carboplatin resulted in an IC_50_ of <0.01 *μ*mol/L (Fig. 5E). Exposure of SCLC line LXFS 650L to LY‐2090314 as a single agent resulted in an IC_50_ of >20 *μ*mol/L and the simultaneous combination of LY2090314 with etoposide/carboplatin produced an IC_50_ of 0.01 *μ*mol/L (Fig. 5F).

**Figure 6:**
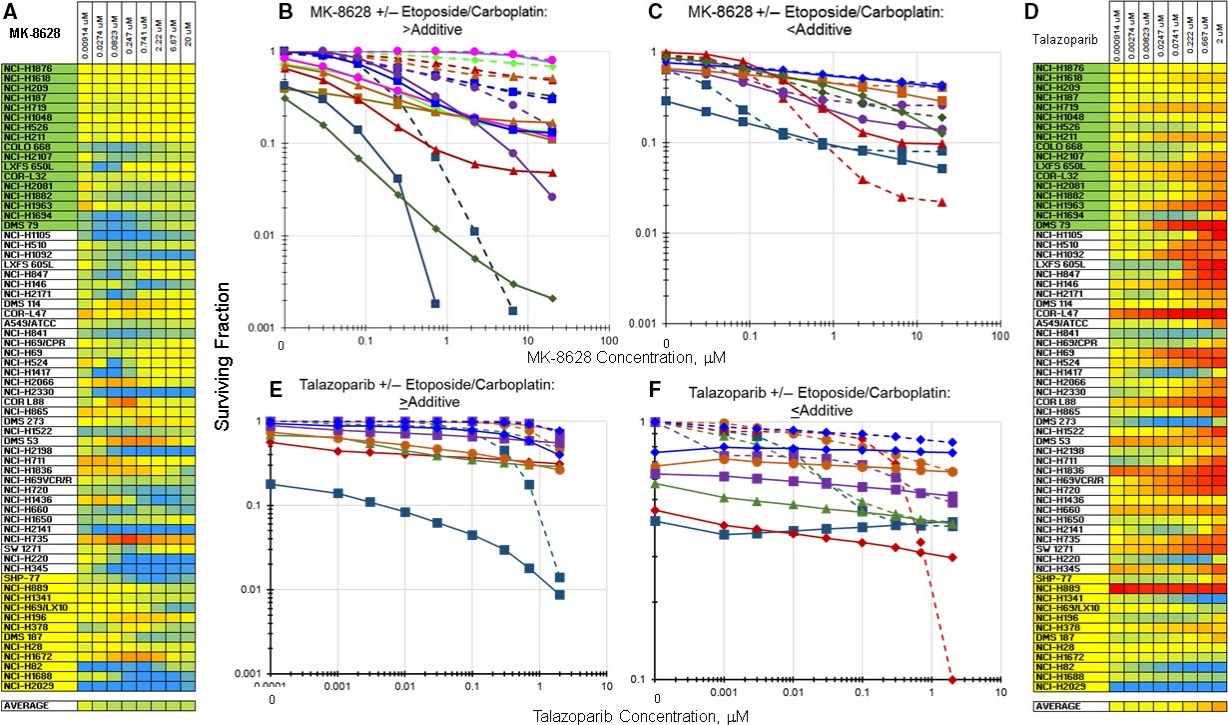
Heatmap and concentration response curves for the addition of the BET bromodomain inhibitor, MK‐8628, or the PARP1 inhibitor, talazoparib, to etoposide/carboplatin in the SCLC lines. (A) Heatmap with the 63 SCLC lines listed from least responsive to most responsive to etoposide. Blue indicates greater than additive response to the combination of MK‐8628 with etoposide/carboplatin (*I*_t_ = −0.281 ± 0.290). Yellow indicates additivity of MK‐8628 with etoposide/carboplatin. Red indicates less than additive response to the combination of MK‐8628 with etoposide/carboplatin. (B) Concentration response curves for representative SCLC lines exposed to MK‐8628 alone (dotted lines), or to the combination of MK‐8628 with etoposide (0.3 *μ*mol/L) and carboplatin (3.7 *μ*mol/L) (solid lines) that produced greater than additive cell killing. (C) Concentration response curves for representative SCLC lines exposed to MK‐8628 alone (dotted lines), or to the combination of MK‐8628 with etoposide (0.3 *μ*mol/L) and carboplatin (3.7 *μ*mol/L) (solid lines) that produced less than additive cell killing. The color of the lines (dotted and solid) indicate the same SCLC line. (D) Heatmap with the 63 SCLC lines listed from least responsive to most responsive to etoposide. Blue indicates greater than additive response to the combination of talazoparib with etoposide/carboplatin. Yellow indicates additivity of talazoparib with etoposide/carboplatin (*I*_t_ = −0.281 ± 0.290). Red indicates less than additive response to the combination of talazoparib with etoposide/carboplatin. (E) Concentration response curves for representative SCLC lines exposed to talazoparib alone (dotted lines), or to the combination of talazoparib with etoposide (0.3 *μ*mol/L) and carboplatin (3.7 *μ*mol/L) (solid lines) that produced greater than additive cell killing. (F) Concentration response curves for representative SCLC lines exposed to talazoparib alone (dotted lines), or to the combination of talazoparib with etoposide (0.3 *μ*mol/L) and carboplatin (3.7 *μ*mol/L) (solid lines) that produced less than additive cell killing. The color of the lines (dotted and solid) indicate the same SCLC line.

Exposure to the BET bromodomain inhibitor MK‐8628 increased the SCLC cell killing by etoposide/carboplatin in 20–25% of the SCLC lines (Fig. 6A). Exposure of the SCLC line NCI‐H345 to MK‐8628 as a single agent produced an IC_50_ of 20 *μ*mol/L. Simultaneous combination of MK‐8628 with etoposide/carboplatin resulted in an IC_50_ of 0.052 *μ*mol/L (Fig. 6B). In other SCLC lines, less effect of adding MK‐8628 to etoposide/carboplatin was observed. Exposure of the SCLC line NCI‐H735 to single agent MK‐8628 produced an IC_50_ of 0.13 *μ*mol/L, while exposure of the NCI‐H735 line to MK‐8628 along with etoposide/carboplatin resulted in an IC_50_ of 0.25 *μ*mol/L (Fig. 6C).

PARP inhibitors have been explored in SCLC both alone and in combination regimens. Among the 63 SCLC panel, 10–15% of the SCLC lines had an increased response to etoposide/carboplatin when simultaneously exposed to the PARP inhibitor talazoparib (Fig. 6D). The DMS 273 SCLC line had a talazoparib IC_50_ of 1.8 *μ*mol/L as a single agent, while the combination of talazoparib with etoposide/carboplatin produced an IC_50_ of 0.014 *μ*mol/L in the DMS 273 SCLC line (Fig. 6E). However, for many SCLC lines, the IC_50_ for talazoparib was ≫2 *μ*mol/L, the highest concentration tested, and the effect of adding talazoparib to etoposide/carboplatin was a modest increase in SCLC killing (Fig. 6F). There is an association between expression of SFLN11 and sensitivity of SCLC lines to etoposide and talazoparib 6, 15. SLFN11 transcript (exon probeset 3753505) had the most consistent association with response to etoposide/carboplatin and to several agents and combinations. Correlation of SLFN11 expression with talazoparib and etoposide/carboplatin had a FDR adjusted *P*‐value of 0.098 for SLFN11 transcript and 0.012 for probeset 3753505 with etoposide/carboplatin; 0.005 for SLFN11 transcript and 0.101 for probeset 3753505 with talazoparib alone; and 0.167 for the whole transcript and *P* = 0.055 for probeset 3753505 with the combination of talazoparib and etoposide/carboplatin, with Pearson's correlation for these tests ranging between −0.430 and −0.627. To identify additional genes involved in response to talazoparib with etoposide/carboplatin, a gene set expression analysis was conducted with SLFN11 transcript and protein expressing SCLC line subset. The gene sets most strongly associated with talazoparib with etoposide/carboplatin encoded a spliceosome complex and RNA splicing via transesterification reaction (LS and KS permutation *P*‐values = 1 × 10^−5^; Efron‐Tibshirani's GSA *P*‐value <0.005) 16, 17.

## Discussion

SCLC is a recalcitrant tumor which responds 60–80% of the time to first‐line therapy and then recurs as a profoundly resistant disease. Genomic studies have confirmed that 75–90% of SCLC has lost TP53 and nearly 100% has lost RB. Other frequent changes include amplification of ASCL1, amplification of MYC, and over expression of NeuroD1 in subsets of SCLC tumors 4, 6, 18, 19, 20. Increased knowledge of the molecular characteristics of SCLC has not yet led to improved therapeutics for this malignancy. Since treatment with the first‐line standard of care agents, etoposide and a platinum complex, can produce good responses, improving the quality of the responses obtained with these agents may result in better long‐term treatment outcomes. This screen examined simultaneous combination exposure of 63 human SCLC lines to etoposide/carboplatin and each of 180 other agents.

It is known that in cell culture, the simultaneous combination of etoposide, a topoisomerase II inhibitor, with paclitaxel, a tubulin stabilizer, produces less than additive cell killing and that the combination of paclitaxel and cisplatin results in additive to less than additive cell killing 21, 22. The current SCLC line screen found that simultaneous combination of taxanes with etoposide/carboplatin resulted primarily in less than additive cell killing. A similar observation applied to the simultaneous combination of vinorelbine, a tubulin fragmenter, with etoposide/carboplatin (Fig. 2). Early phase clinical trials examined the efficacy of a platinum complex with etoposide and vinorelbine in non‐small cell lung cancer 23, 24, 25. In these early studies, the platinum complex and etoposide were administered on similar schedules and vinorelbine was administered intermittently and all three drugs were administered intravenously. The regimen had some activity but was toxic. More recently, a randomized trial in SCLC was reported in which etoposide/cisplatin was alternated with vinorelbine/cisplatin and compared with etoposide/cisplatin 24. The trial found that the etoposide/cisplatin, vinorelbine/cisplatin alternating regimen prolonged progression‐free survival of SCLC patients compared with the etoposide/cisplatin regimen.

Inhibitors of aurora kinase, KSP/Eg5 kinase, and polo‐like‐1 kinase were found to be active agents in a large single‐agent screen of 63 human SCLC lines 6. Each of these classes of agents has a role during mitosis. Ten aurora kinase inhibitors were tested in the 63 SCLC line single‐agent screen and most were active in 30–40% of the lines. However, in combination with etoposide/carboplatin, in a screen with the same human SCLC lines, alisertib produced primarily less than additive cell killing. In a panel of 23 SCLC lines, the aurora kinase inhibitor barasertib (AZD1152) was most active in lines with cMYC amplification or high cMYC expression 26. In a CTEP‐sponsored phase II single‐agent clinical trial in sarcoma, alisertib was well‐tolerated and produced a promising progression‐free survival but missed the primary response rate endpoint 27. Two clinical trials included SCLC patients. A phase II study of alisertib in combination with paclitaxel versus paclitaxel alone (NCT02038647) has completed patient accrual. Another clinical trial, alisertib along with paclitaxel in East Asian patients with advanced solid tumors including SCLC (NCT02367352), is ongoing.

Kinesin spindle protein (KSP/Eg5) is a motor protein that functions during mitosis to translate ATP hydrolysis into mechanical force, thus driving the organization of the mitotic spindle 28. The KSP/Eg5 inhibitor ispinesib prevents the release of ADP from the KSP protein, thus preventing spindle pole separation. Ispinesib was active across a panel of 23 breast cancer lines and produced regression in five breast cancer xenografts 29. Ispinesib has undergone phase 1 clinical trial in advanced breast cancer and limited activity was observed 30. Ispinesib was studied in several Phase II clinical trials, however, there was insufficient activity to move on. In the 63 SCLC line, single‐agent screen, 6 KSP inhibitors were tested and most had good activity in the SCLC lines. However, in the 63 SCLC line combination screen, ispinesib in simultaneous combination with etoposide/carboplatin produced less than additive cell killing 6 (Fig. 3).

High expression of polo‐like kinase‐1 (PLK1), a protein which has multiple functions during mitosis, has been associated with poor prognosis in several tumor types. GSK461364 is a substrate for the ABCB1 efflux pump which limits activity in some preclinical models 31, 32. Seven polo‐like kinase 1 inhibitors were tested in the single agent 63 SCLC screen and had activity in 60–70% of the SCLC lines. The Polo‐like kinase 1 inhibitor GSK461364 underwent phase I clinical trial in patients with advanced solid malignancies 33, 34. However, an unexpected adverse event, venous thrombotic emboli of grade 3–4, occurred in some patients. A phase II dose was determined and co‐administration of an anticoagulant was recommended. Several polo‐like kinase inhibitors are undergoing phase II clinical trials. GSK‐461364 was a potent single agent in a subset of the SCLC lines; however, simultaneous combination of GSK‐461364 with etoposide/carboplatin produced less than additive SCLC killing (Fig. 3) 6.

Among the agents which increased the SCLC killing with etoposide/carboplatin was the MDM2 inhibitor JNJ‐27291199. JNJ‐27291199 was an early MDM2 inhibitor. It is less potent and perhaps less selective than more current MDM2 inhibitors 35, 36, 37. Theoretically, only tumors with wild‐type (WT) TP53 can potentially be sensitive to TP53‐MDM2 inhibitors and clinical trials of TP53‐MDM2 inhibitors only include patients with WT TP53 tumors 38, 39, 40. Other MDM2 binding partners such as XIAP mRNA have been identified 41. JNJ‐27291199 is not an active agent in SCLC lines, and the increased cytotoxicity observed with JNJ‐27291199 in combination with etoposide/carboplatin in the SCLC lines may be due to an alternate target of the compound (Fig. 4). There are about 20 ongoing clinical trials exploring the clinical benefit of MDM2 inhibitors (ClinicalTrials.gov).

Following DNA damage, CHK1 prevents entry of cells into mitosis and coordinates DNA repair; thus, inhibition of CHK1 can sensitize proliferating tumor cells to DNA damaging agents 42, 43. CHK1 is a regulator of the G2/M checkpoint with a role in replication fork stability and homologous recombination. Although some CHK1 inhibitors tend to have limited single‐agent activity likely due to compensation by activation of ATM and ERK1/2 pathway, these agents are being developed as single agent therapeutics 44, 45. LY2603618 is a potent, selective CHK‐1 inhibitor 46. In cell‐based assays and in human tumor xenografts, LY2603618 enhanced the activity of anticancer drugs but had modest single‐ agent activity 47. However, in combination regimens of LY2603618 with pemetrexed or gemcitabine there was a greater tumor response than with either chemotherapeutic agent alone. In a phase 1 clinical study, LY2603618 administered as a 1‐h infusion approximately 24 h after pemetrexed had acceptable safety and pharmacokinetics 48. There are several clinical trials ongoing with CHK‐1 inhibitors in a variety of tumor types (ClinicalTrials.gov). Among them are phase 2 trials with the next‐generation CHK‐1 inhibitor prexasertib as a single agent in BRCA1/2 mutation‐associated breast or ovarian cancer and in patients with solid tumors with replicative stress or homologous repair deficiency.

Glycogen synthase kinase‐3 (GSK3) is involved in many intracellular signaling pathways 49. GSK3 has been known since the 1970s and has been implicated in a wide range of conditions including CNS pathologies, diabetes, and cancer 50, 51. In cancer, GSK3*β* has been shown to be involved in the generation of cancer stem cells. The Wnt‐*β*‐catenin signaling pathway is activated by GSK3*β* because GSK3*β* controls the stability and degradation of *β*‐catenin 52. The Wnt‐GSK3‐*β*‐catenin pathway is frequently associated with tumorigenesis. In cancer cells, GSK3*β* inhibition alone or in combination with a DNA‐damaging agent can induce prosurvival, autophagic signals 53, 54, 55, 56. LY2090314 is a GSK3*β* inhibitor which had preclinical efficacy in human tumor xenografts when combined with platinum‐based regimens 57. Furthermore, LY2090314 has undergone a first‐in‐human phase 1 trial in combination with pemetrexed and carboplatin. There were 11 dose‐limiting toxicities reported including thoracic pain which was controlled with ranitidine. The trial reported five confirmed partial responses and 19 stable disease 58. A phase 2/3 clinical trial of LY2090314 in combination with gemcitabine and nab‐paclitaxel was terminated (NCT01632306). LY2090314 had little or no activity in the SCLC lines as a single agent. However, in a subset of the SCLC lines, LY2090314 in combination with etoposide/carboplatin produced greater than additive cell killing (Fig. 5).

Among epigenetic targets, BET bromodomains appear promising. Bromodomain‐containing proteins bind to acetyl‐lysines on acetyl‐dependent transcriptional regulator complexes. BET bromodomains (BRD2, BRD3, BRD4, and BRDT) are druggable targets 58, 59, 60. The BET bromodomain inhibitor MK‐8628 had single agent activity in a few SCLC lines and in select SCLC lines enhanced the activity of etoposide/carboplatin (Fig. 6). There was a trend for MYC amplified SCLC lines to be more sensitive to MK‐8628 than other SCLC lines, however, this effect did not reach significance 9. There are about 20 clinical trials of BET bromodomain inhibitors underway (ClinicalTrials.gov). The combination of the PARP1 inhibitor talazoparib with etoposide/carboplatin produced highly mixed results with a subset of SCLC lines having enhanced cytotoxicity with the combination and another subset having decreased cytotoxicity with the combination (Fig. 6D). There is a growing literature indicating that the protein SFLN11 is involved in the response of cells to DNA damaging agents such as etoposide and carboplatin and with the PARP inhibitors 61, 62, 63, 64, 65. SLFN11 gene and exons expression correlated with sensitivity to etoposide/carboplatin, to talazoparib and to talazoparib with etoposide/carboplatin; however, both the talazoparib/etoposide/carboplatin responsive and nonresponsive SCLC lines expressed SLFN11. By gene set expression comparison analysis, very subtle differences were seen in spliceosome gene expression and RNA splicing by transesterification reaction pathways between responsive and nonresponsive SCLC lines 10, 11. Talazoparib is in about 25 clinical trials including at least one trial which includes SCLC patients (ClinicalTrials.gov).

SCLC remains a highly recalcitrant disease. Newer therapeutics including antibody drug conjugates, immunotherapeutics, and new targeted drugs are being tested in SCLC patients. Because recurrent SCLC is remarkably therapeutically resistant, one strategy to improving patient outcome may be increasing the therapeutic benefit from first‐line chemotherapy. In our studies, a few newer agents could increase SCLC killing in cell culture when added to etoposide/carboplatin. A next step may be to test these agents in tumor‐bearing mice to assess the potential benefit of the combinations in vivo.

## Conflict of Interest

None declared.

## Supporting information


**Figure S1.** Heatmap showing the results of the SCLC combination screen. The SCLC lines were listed from least responsive to etoposide/carboplatin to most responsive to etoposide/carboplatin. Blue indicates combinations that were greater than additive, white indicates combinations that appeared to be additive and red indicates combinations that were less than additive.

**Figure S2.** Heatmap and concentration response curves for agents that produced mainly less than additive SCLC killing in combination with etoposide/carboplatin. Panel A: IC_50_ heatmap for the 63 SCLC lines from an 8‐point concentration response screen performed in triplicate in concentrations ranging from 10 *μ*mol/L to 0.0015 *μ*mol/L exposed to paclitaxel, vinorelbine, alisertib, ispinesib, or GSK‐461364 for 96 h. Red indicates potent cell killing and green indicated no cell killing. Panel B: Concentration response curves for the 63 SCLC lines exposed to paclitaxel, vinorelbine, alisertib, ispinesib, or GSK‐461364 for 96 h.

**Figure S3**. Heatmaps for the addition of a nuclear kinase inhibitor to etoposide/carboplatin in the SCLC lines. Left: Heatmap with the 60 SCLC lines listed from least responsive to most responsive to etoposide. Yellow indicates additivity of the aurora kinase inhibitor, alisertib, with etoposide/carboplatin. Red indicates less than additive response to the combination of alisertib with etoposide/carboplatin. Center: Heatmap with the 60 SCLC lines listed from least responsive to most responsive to etoposide. Yellow indicates additivity of the KSP/EG5 inhibitor, ispinesib, with etoposide/carboplatin. Red indicates less than additive response to the combination of ispinesib with etoposide/carboplatin. Right: Heatmap with the 60 SCLC lines listed from least responsive to most responsive to etoposide. Yellow indicates additivity of the Polo‐like kinase inhibitor, GSK‐461364, with etoposide/carboplatin. Red indicates less than additive response to the combination of GSK‐461364 with etoposide/carboplatin.

**Figure S4.** Heatmap and concentration response curves for agents that produced mainly less than additive SCLC killing in combination with etoposide/carboplatin. Panel A: IC_50_ heatmap for the 60 SCLC lines from an 8‐point concentration response screen performed in triplicate in concentrations ranging from 10 *μ*mol/L to 0.0015 *μ*mol/L exposed to LY‐2090314, rabusertib, JNJ‐27291199, talazoparib, or JQ1 for 96 h. Red indicates potent cell killing and green indicated no cell killing. Panel B: Concentration response curves for the 60 SCLC lines exposed to LY‐2090314, rausertib, JNJ‐27291199, talazoparib, or JQ1 for 96 h.

**Figure S5**. Heatmap including 20 SCLC lines that express high SLFN11 transcript and protein, 14 are responsive to talazoparib plus etoposide/carboplatin and 6 do not respond to talazoparib plus etoposide/carboplatin. Expression of 7 transcripts with original *P* < 0.0002 and FDR‐adjusted *P* < 0.4 with a median log_2_ gene expression >6 could distinguish responsive from unresponsive SCLC lines exposed to talazoparib simultaneously with etoposide/carboplatin.
