# Supplementary figures and images for: Small cell lung carcinoma cell line screen of etoposide/carboplatin plus a third agent
Source: Cancer Med. 2017 Aug 1;6(8):1952–64. doi: 10.1002/cam4.1131 (PMC5548882; doi:10.1002/cam4.1131)

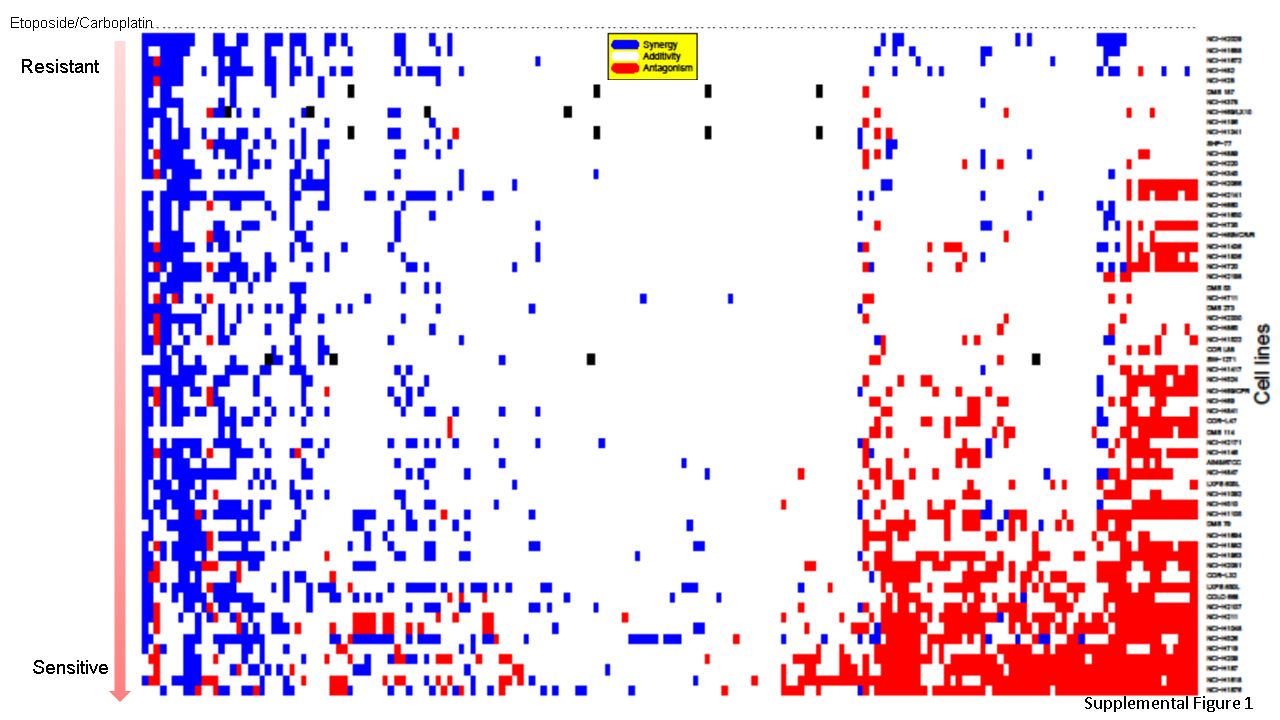

Supplement: Supplementary file 1 — Figure S1. Heatmap showing the results of the SCLC combination screen. The SCLC lines were listed from least responsive to etoposide/carboplatin to most responsive to etoposide/carboplatin. Blue indicates combinations that were greater than additive, white indicates combinations that appeared to be additive and red indicates combinations that were less than additive. [file CAM4-6-1952-s001.jpg]

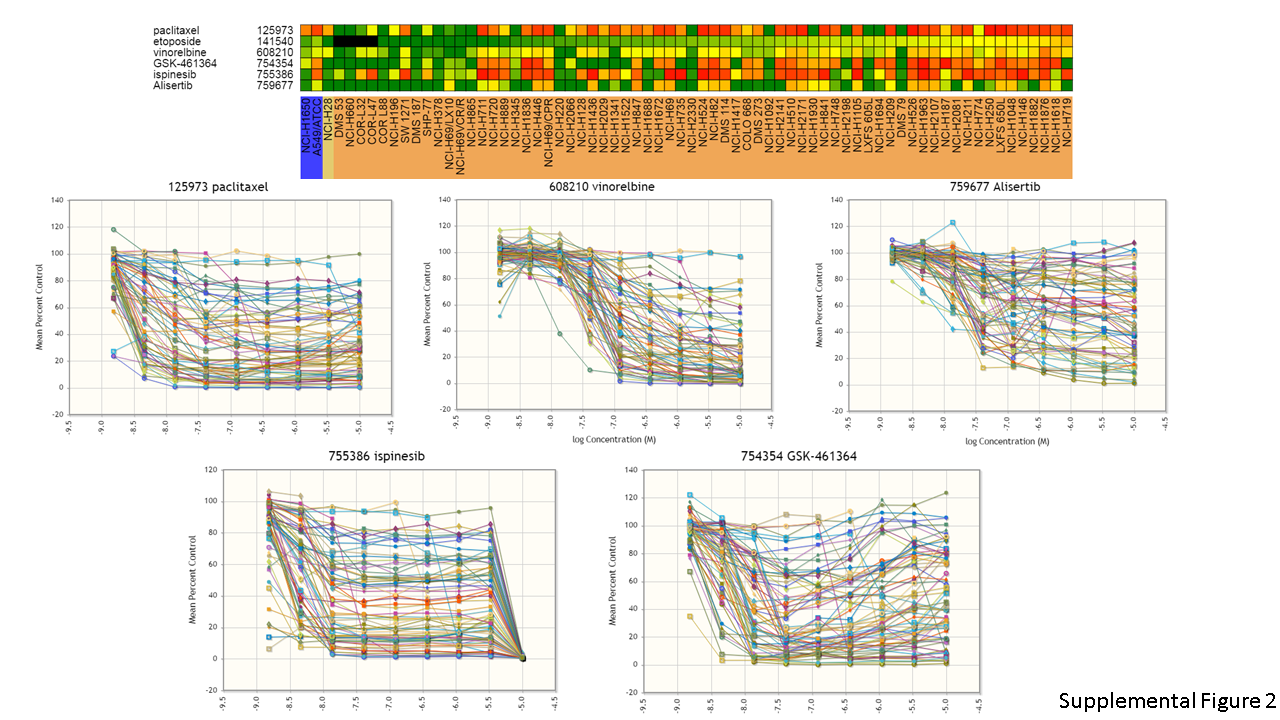

Supplement: Supplementary file 2 — Figure S2. Heatmap and concentration response curves for agents that produced mainly less than additive SCLC killing in combination with etoposide/carboplatin. Panel A: IC50 heatmap for the 63 SCLC lines from an 8‐point concentration response screen performed in triplicate in concentrations ranging from 10 μmol/L to 0.0015 μmol/L exposed to paclitaxel, vinorelbine, alisertib, ispinesib, or GSK‐461364 for 96 h. Red indicates potent cell killing and green indicated no cell killing. Panel B: Concentration response curves for the 63 SCLC lines exposed to paclitaxel, vinorelbine, alisertib, ispinesib, or GSK‐461364 for 96 h. [file CAM4-6-1952-s002.png]

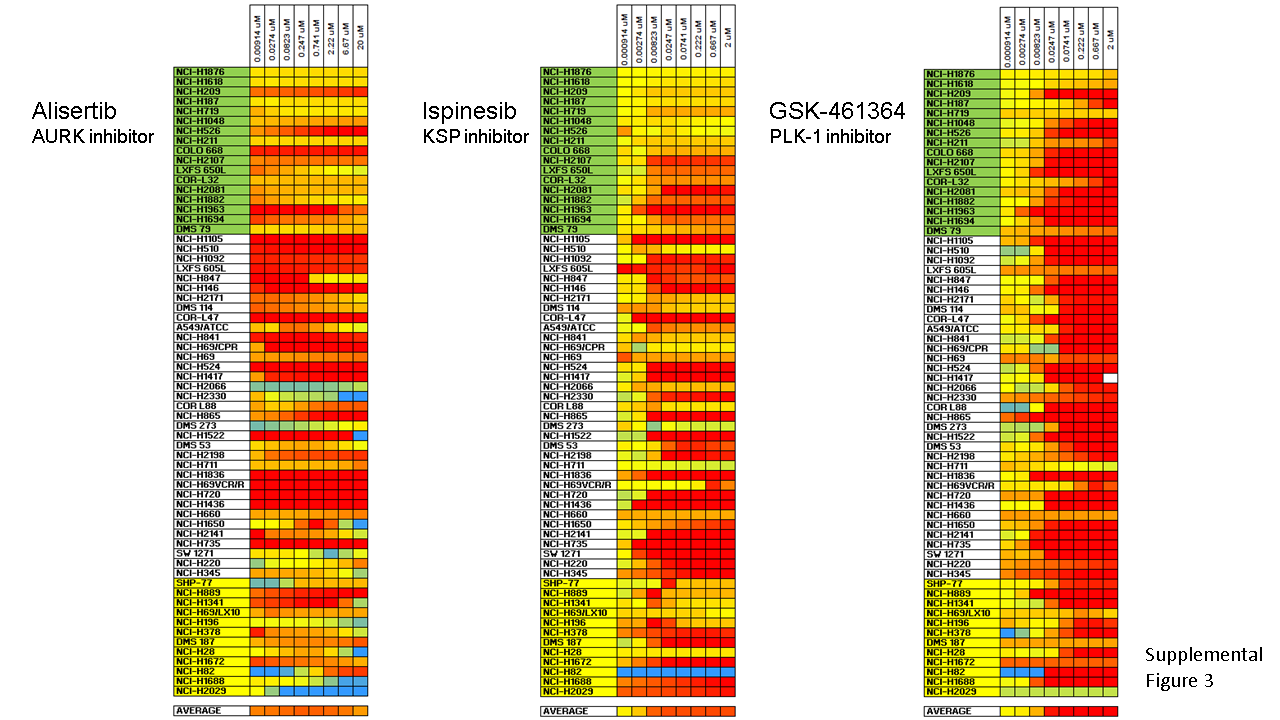

Supplement: Supplementary file 3 — Figure S3. Heatmaps for the addition of a nuclear kinase inhibitor to etoposide/carboplatin in the SCLC lines. Left: Heatmap with the 60 SCLC lines listed from least responsive to most responsive to etoposide. Yellow indicates additivity of the aurora kinase inhibitor, alisertib, with etoposide/carboplatin. Red indicates less than additive response to the combination of alisertib with etoposide/carboplatin. Center: Heatmap with the 60 SCLC lines listed from least responsive to most responsive to etoposide. Yellow indicates additivity of the KSP/EG5 inhibitor, ispinesib, with etoposide/carboplatin. Red indicates less than additive response to the combination of ispinesib with etoposide/carboplatin. Right: Heatmap with the 60 SCLC lines listed from least responsive to most responsive to etoposide. Yellow indicates additivity of the Polo‐like kinase inhibitor, GSK‐461364, with etoposide/carboplatin. Red indicates less than additive response to the combination of GSK‐461364 with etoposide/carboplatin. [file CAM4-6-1952-s003.png]

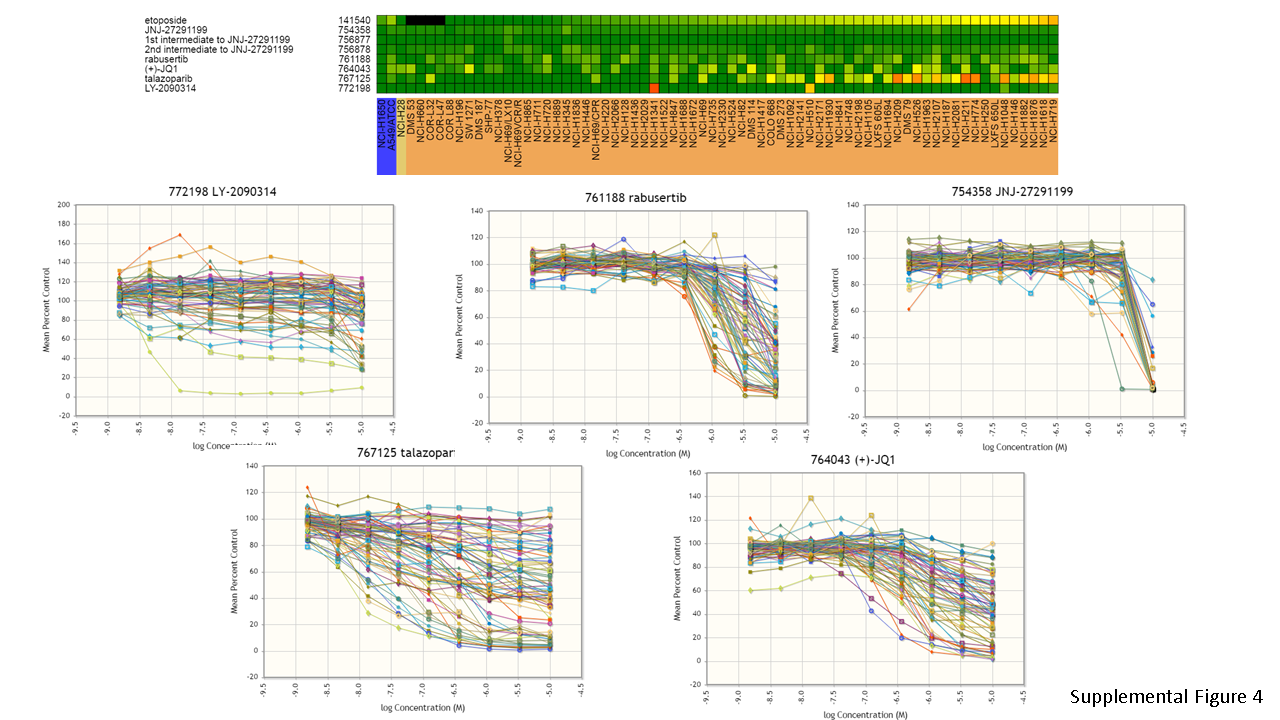

Supplement: Supplementary file 4 — Figure S4. Heatmap and concentration response curves for agents that produced mainly less than additive SCLC killing in combination with etoposide/carboplatin. Panel A: IC50 heatmap for the 60 SCLC lines from an 8‐point concentration response screen performed in triplicate in concentrations ranging from 10 μmol/L to 0.0015 μmol/L exposed to LY‐2090314, rabusertib, JNJ‐27291199, talazoparib, or JQ1 for 96 h. Red indicates potent cell killing and green indicated no cell killing. Panel B: Concentration response curves for the 60 SCLC lines exposed to LY‐2090314, rausertib, JNJ‐27291199, talazoparib, or JQ1 for 96 h. [file CAM4-6-1952-s004.png]

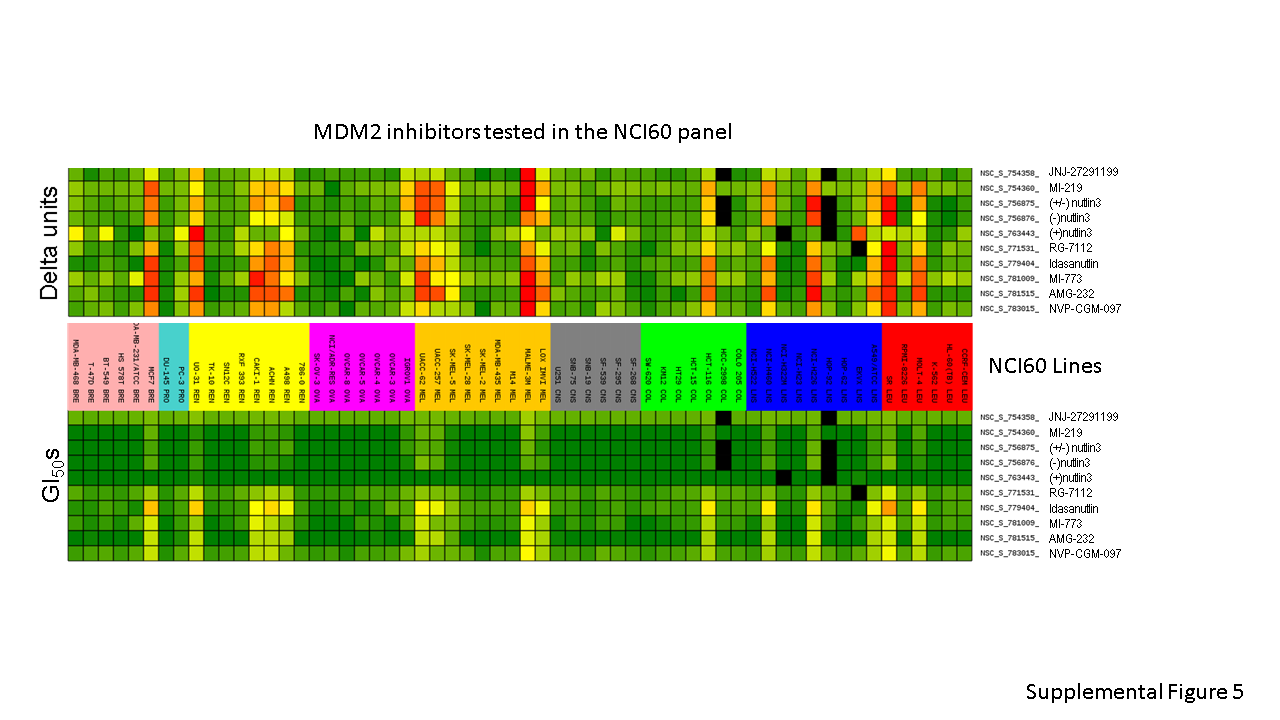

Supplement: Supplementary file 5 — Figure S5. Heatmap including 20 SCLC lines that express high SLFN11 transcript and protein, 14 are responsive to talazoparib plus etoposide/carboplatin and 6 do not respond to talazoparib plus etoposide/carboplatin. Expression of 7 transcripts with original P < 0.0002 and FDR‐adjusted P < 0.4 with a median log2 gene expression >6 could distinguish responsive from unresponsive SCLC lines exposed to talazoparib simultaneously with etoposide/carboplatin. [file CAM4-6-1952-s005.png]
